# Case Report: A long-term survival case of diffuse large B-cell lymphoma with left ventricular infiltration and spinal cord compression

**DOI:** 10.3389/fcvm.2023.1309613

**Published:** 2023-12-20

**Authors:** Rui Sun, Chenxing Jin, Henan Qin, Wenhe Zhang, Zhen Ning, Jiwei Liu, Aman Wang

**Affiliations:** ^1^ Department of Oncology, The First Affiliated Hospital of Dalian Medical University, Dalian, China; ^2^ Department of General Surgery, The First Affiliated Hospital of Dalian Medical University, Dalian, China

**Keywords:** cardiac lymphoma, cardiac mass, intracardiac mass, lymphoma, large B-cell, diffuse

## Abstract

**Background:**

Diffuse large B-cell lymphoma (DLBCL) is the most common subtype of non-Hodgkin lymphoma and may occur with lymph node and/or extranodal involvement. However, DLBCL with intracardiac mass is exceedingly rare. In the reported literature, the intracardiac infiltration of DLBCL mostly involves the right ventricle. Lymphoma that invades the heart has an aggressive nature, with symptoms that are easily ignored initially and can lead to multiple complications in severe cases, resulting in a poor prognosis. Early screening and diagnosis may significantly improve the survival rate. Early diagnosis may significantly improve outcomes.

**Case summary:**

We presented a 68-year-old woman with back pain. PET/CT suggested increased FDG metabolism in the left ventricle, right adrenal gland, right erector spinae intramuscularis, multiple bones and multiple lymph nodes. Contrast-enhanced ultrasound showed a left ventricular apical mass with ventricular septum thickening. Cardiac MRI suggested a 1.6*1.1*2.1 cm mass in the apical-central portion of the left ventricle. Biopsy of the right neck mass confirmed the pathologic diagnosis of diffuse large B-cell lymphoma. However, before the pathologic diagnosis was confirmed, the patient was paralyzed due to spinal cord compression caused by the progression of bone metastases. Subsequently, pathology confirmed the diagnosis of diffuse large B-cell lymphoma, and rituximab plus cyclophosphamide, doxorubicin, vincristine, and prednisone (R-CHOP) was treated immediately as first-line therapy. In addition, glucocorticoids and mannitol dehydration were administered to relieve the symptoms of spinal cord compression. After 8 cycles of R-CHOP, the tumor at all sites had almost complete regression. The patient was able to walk normally and had no tumor-related symptoms.

**Conclusions:**

We present a case of DLBCL with a very high tumor load that involved multiple organs, including the left ventricle, but exhibited no cardiac-related symptoms. The combination of various imaging modalities is valuable for the diagnosis of cardiac infiltration. The mass in the left ventricle almost completely regressed after R-CHOP treatment, and no recurrence has occurred in the 5 years of follow-up so far.

## Introduction

Lymphoma-derived intracardiac masses are more common in two types: primary cardiac lymphoma (PCL) and cardiac invasion by extracardiac lymphoma (1). PCL is an extranodal lymphoma that often involves the right side of the heart. In non-PCL lymphomas, DLBCL has been reported to involve the heart, but left ventricular infiltration is extremely rare. Primary cardiac lymphoma is rare, accounting for less than 2% of all resected primary cardiac tumors in autopsy, and 0.5% of extranodal lymphomas. However, secondary cardiac involvement exists in 25% of patients with disseminated lymphoma (1).

## Case presentation

A 62-year-old woman presented to our hospital with lower back pain for 3 months. She had no history of fever, night sweats, weight loss or other symptoms. She had a 9-year history of hypertension, with a maximum blood pressure of 160/100 mmHg, which was controlled at about 120/80 mmHg with amlodipine benzenesulfonate. She had no history of other underlying conditions including coronary artery disease, chronic heart failure, and so on. On admission, her vital signs were stable with a blood pressure of 130/80 mm Hg, a heart rate of 102 beats/min and a temperature of 36.3°C. Because the severe low back pain interfered with sleep, she was mostly confined to bed and was treated with opioid analgesia. A 3.0 cm × 3.0 cm mass was palpable in the right supraclavicular region of the patient, with firm texture, poor mobility, and no tenderness. There was tenderness and percussion pain (+) in the 4, 8, and 11 thoracic regions. Her cardiovascular exam was normal. Laboratory tests revealed moderately elevated lactate dehydrogenase (LDH) at 476 IU/L (100–300 IU/L) and elevated β2 microglobulin (β2MG) at 2.54 mg/L(0–0.3 mg/L). The hematuria and stool routine, liver and kidney function, electrolytes, and tumor markers were normal. The electrocardiogram showed a sinus rhythm with a left axis deviation.

**Figure 1:**
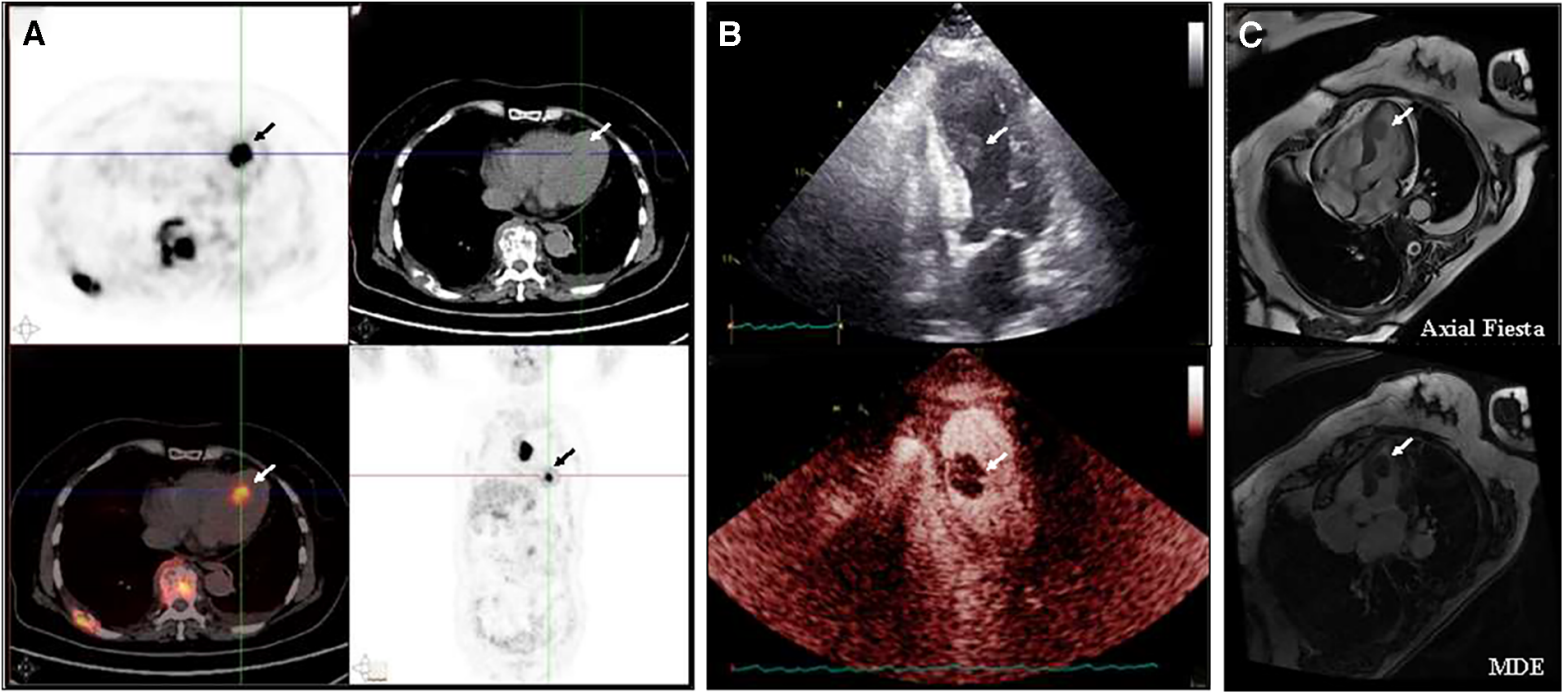
Baseline imaging of the left ventricular mass (**A**) PET/CT indicated multiple masses in the left ventricle, gallbladder, right adrenal gland, and right vertebral column, along with significant lymph node enlargement and widespread bone destruction, all exhibiting markedly increased FDG uptake. (**B**) Echocardiography showed an additional echogenicity measuring 14 × 17 mm in the proximal segment of the left ventricular septum, with an irregular shape, tissue-like echogenicity, and activity. A detectable filling defect in the septum near the apex is observed, with an irregular shape, approximately 12 × 22 mm in size, broad at the base, highly mobile at the attachment point, and containing a relatively large amount of contrast filling material. (**C**) CMR showed a mass measuring 1.6 cm × 1.1 cm × 2.1 cm at the left cardiac chamber septum. Long-axis four-chamber myocardial delayed enhancement (MDE) showed that the left ventricular mass appeared non-gadolinium enhancement at baseline.

To determine whether the masses were malignant, positron emission tomography (PET) imaging was performed with ${}^{18}F\text{-}\mathrm{fluorodeoxyglucose}\;(\mathrm{FDG})$. The PET imaging showed multiple masses in the left ventricle, gallbladder, right adrenal gland, and right erector spinae with multiple enlarged lymph nodes and bone destruction throughout the body, all of which had significantly increased FDG uptake. In particular, the maximum standardized uptake value for left ventricular masses was 16.5 and 19.7 after delay, consistent with malignancy (Figure 1A). Echocardiography showed a 14 × 17 mm additional echo detectable at the proximal apical segment of the septum in the left ventricle, with irregular morphology, tissue-like echoes, and mobility. Echocardiography of the cardiac chambers showed a detectable contrast-enhanced mass in the septum near the apex, with an irregular morphology, measuring approximately 12 × 22 mm, with a wide base and high mobility at the point of attachment, and with a relatively large amount of contrast filling detectable within it, which was considered to be malignant (Figure 1B). The left ventricular ejection fraction (LVEF) was 59%. Axial Fiesta of cardiac magnetic resonance (CMR) showed a mass measuring 1.6 cm × 1.1cm × 2.1 cm at the left cardiac chamber septum. Long-axis four-chamber myocardial delayed enhancement (MDE) showed nodular shadows in the left cardiac chamber, and the mass had no gadolinium enhancement (Figure 1C). The patient refused to undergo an endomyocardial biopsy because of concerns about the risks of invasive procedures. Then, the patient underwent a biopsy of the right supraclavicular lymph node.

However, before the pathologic diagnosis was confirmed, the patient experienced spinal cord compression due to bone metastasis. Physical examination indicated that pain and temperature sensation below the level of the fourth thoracic vertebra disappeared, muscle strength of both lower limbs was grade 2, and no pathological reflexes were elicited. Magnetic resonance of the spine suggests bony destruction of vertebrae and appendages of thoracic vertebrae 4, 5, and 12, surrounded by visible soft tissue masses with spinal cord compression and edema. We hypothesized that the patient presented with spinal cord compression due to bone metastases. Glucocorticoids and mannitol dehydration were administered immediately to relieve the symptoms of spinal cord compression. Subsequently, pathology confirmed the diagnosis of diffuse large B-cell lymphoma, with positive immunohistochemical staining for CD20, CD3, CD5, BCL-2, and Ki67 (50%+).

**Figure 2:**
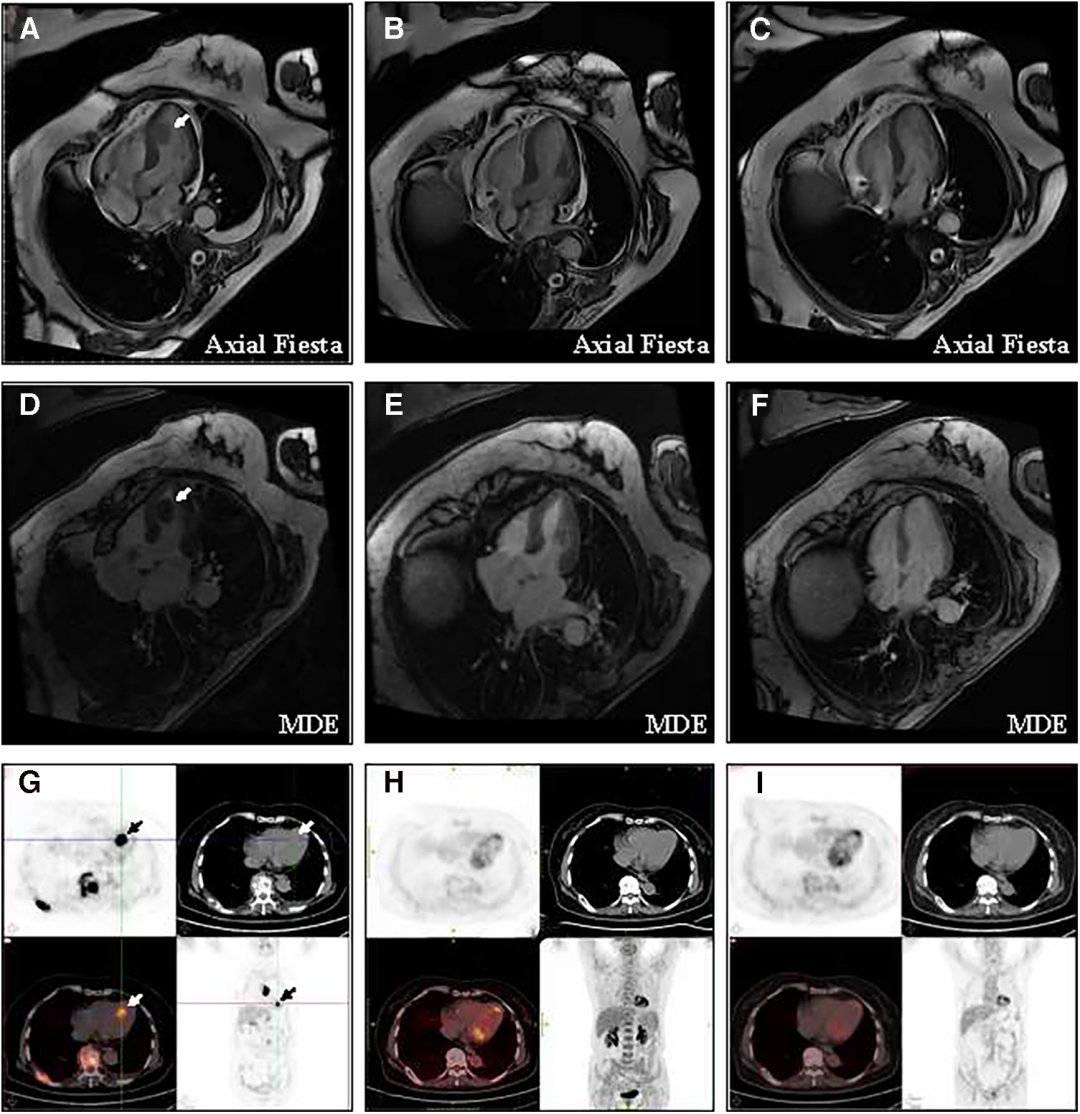
Change of the left ventricular mass at baseline and after treatment (**A**) CMR showed that the baseline size of the mass is 1.6 cm × 1.1 cm × 2.1 cm. (**B**) CMR showed that after 4 cycles of R-CHOP, the tumor at all sites had almost complete regression. (**C**) CMR showed no recurrence of cardiac mass in the five years of follow-up. (**D**) Long-axis four-chamber myocardial delayed enhancement (MDE) showed that the left ventricular mass appeared non-gadolinium enhancement at baseline. (**E**) After 4 cycles of R-CHOP treatment, long-axis four-chamber MDE showed the left ventricular mass had almost complete regression. (**F**) MDE showed no recurrence of cardiac mass in the five years of follow-up. (**G**) PET/CT showed that the maximum standardized uptake value for left ventricular masses was 16.5 and 19.7 after delay. (**H**) After 4 cycles of R-CHOP treatment, the left ventricle mass had almost complete regression and the FDG uptake of the left ventricular had returned to normal levels. (**I**) PET/CT showed no recurrence of cardiac mass in the five years of follow-up.

Rituximab plus cyclophosphamide, doxorubicin, vincristine, and prednisone (R-CHOP) was treated immediately as first-line therapy. Almost all sites of tumor lesions were significantly reduced after two cycles of R-CHOP. The left ventricular mass was reduced to 1.1 cm × 0.6 cm, and the pain and temperature sensation below the level of the 4th thoracic vertebra were normalized, and the muscle strength of the left and right lower limbs improved to levels 4 and 5, respectively. After 4 cycles of R-CHOP, the left ventricular mass had almost regressed and the muscle strength of both lower limbs had completely returned to normal. A total of 6 cycles of R-CHOP were completed for first-line treatment, and the efficacy evaluation was complete response and then follow-up. During the treatment period, the LVEF increased from 65% at baseline to 69%, and no cardiovascular-related adverse events were observed. During the 5-year follow-up, all lesions, including the cardiac mass, remained complete regression, and FDG uptake was reduced to normal (Figure 2). The patient was able to walk normally and had no tumor-related symptoms. At the time of this manuscript (more than 5 years from follow-up), she is alive and has normal functional capacity. Despite the patient receiving anthracycline-based chemotherapy in first-line treatment, the LVEF remained normal, and no long-term cardiovascular toxicity was observed at 5 years of follow-up.

## Discussion

The nature of cardiac masses is mostly attributed to intracardiac thrombi and benign mesenchymal tumors, while malignant tumors are rare, making their differential diagnosis challenging (2). Malignant cardiac tumors can be categorized by tissue type into sarcomas, mesotheliomas, and lymphomas, with sarcomas being the most common (2). Lymphomas presenting as intracardiac masses are exceedingly rare and can either originate within the heart as primary cardiac lymphomas or infiltrate the heart from extracardiac lymphomas. Infiltration of the heart by extracardiac lymphomas is more common, particularly in immunocompromised patients (3). Clinical manifestations of lymphoma-induced intracardiac masses lack specificity but commonly include symptoms such as dyspnea, lower limb edema, signs of right heart failure, intracardiac thrombi, peripheral emboli, valvular heart disease, and pericardial effusion. B-symptoms such as weight loss, night sweats, and fever may also be present (3). Lymphomas most frequently involve the right ventricle and the pericardium, with isolated involvement of the left ventricle being exceptionally rare. The precise mechanism leading to cardiac tropism in lymphomas remains unclear (3). In some cases, when tumors invade the heart, acute signs can precede B-symptoms (occurring in less than 25% of patients), aiding in early lymphoma diagnosis, with a reported incidence of acute heart failure being 20% (4). In our reported case of DLBCL, the intracardiac mass was located in the left ventricular apex-central region and did not manifest cardiac-related symptoms, making it an extremely rare clinical presentation. However, previous studies reported that left ventricular mass may increase the risk of embolization (5, 6). Although the cardiac mass of this patient was asymptomatic, the necessity for prophylactic anticoagulation is worth discussing.

Histopathological biopsy is the gold standard for lymphoma diagnosis. For patients with palpable superficial lymph node enlargement, lymph node biopsy is the preferred and less invasive option. In cases where the nature of intracardiac masses is difficult to determine, some experts suggest considering endocardial biopsy for diagnosis, although this procedure carries significant invasive risks (7). Transthoracic echocardiography is a non-invasive, cost-effective method for cardiac examination. However, it has limitations in specific views, and image quality and tissue resolution depend on the operator's experience (8). Cardiac CT offers advantages of high spatial resolution and three-dimensional volume acquisition, making it valuable for assessing the anatomical morphology and calcification of masses. It is especially useful for evaluating masses or tumors close to or invading the coronary arteries or bypass grafts. However, it has low tissue contrast, making it less suitable for delineating tumor borders (1). MRI provides high temporal resolution and tissue contrast, aiding in qualitative analysis of tumors. Both primary and secondary lymph nodes exhibit equal T1 and long T2 signals on MRI (9). PET/CT, as a sensitive whole-body imaging technique, plays a significant role in differentiating the benign and malignant nature of cardiac masses, assessing overall tumor burden, and predicting prognosis. Previous studies have shown that the majority of cardiac lymphomas are visible on PET/CT (9). Dynamic monitoring with PET/CT after treatment helps evaluate residual tumors and treatment efficacy, optimizing follow-up care. In our case, cardiac ultrasound and cardiac MRI both supported the diagnosis of a malignant tumor, which was confirmed by high-intensity FDG uptake on PET imaging. Due to concomitant superficial lymph node enlargement, a biopsy of the right cervical lymph node confirmed DLBCL, eliminating the need for invasive endocardial biopsy.

DLBCL is an aggressive lymphoma with rapid disease progression, and DLBCL involving the heart carries a poor prognosis. Without treatment, only 10% of patients survive for 9–12 months (10, 11). Clinical aggressiveness is consistent with known factors such as high proliferation index, activated B-cell lymphoma phenotype, and expression or rearrangement of MYC, which are associated with extranodal sites and poorer outcomes (12). However, DLBCL is sensitive to treatment. The first-line R-CHOP regimen has shown favorable response rates, with standardized chemotherapy regimens achieving response rates of up to 79%–87% (4). The heart may not be considered a sanctuary for systemic drugs. Currently, there is no evidence to suggest that lymphomas in the cardiac location increase the risk of cardiotoxicity from chemotherapy, especially anthracyclines or rituximab, and this remains inconclusive. Early relapse or refractory disease may be managed with additional chemotherapy and allogeneic stem cell transplantation. In our patient, after R-CHOP chemotherapy, nearly complete regression of systemic masses was achieved, which is expected for diffuse large B-cell lymphoma.

Current guidelines recommend routine follow-up every 3–6 months for effectively treated patients, including medical history review, physical examination, and laboratory tests for 5 years. Imaging follow-up should not exceed every 6 months for 2 years unless clinically indicated (13). Our patient, after 5 years of follow-up post-treatment, has shown no tumor recurrence and remains free of any tumor-related symptoms, leading a normal daily life.

Our case represents a rare occurrence of diffuse large B-cell lymphoma involving the left ventricular apex-central region without cardiac-related symptoms, making it an exceptionally rare clinical presentation. The patient underwent multiple cardiac imaging modalities, highlighting the importance of a multidisciplinary approach in the differential diagnosis of cardiac tumors. The patient demonstrated significant efficacy following chemotherapy combined with targeted therapy, resulting in nearly complete tumor regression and over 5 years of tumor-free follow-up. Early diagnosis and treatment are key to achieving favorable outcomes in lymphoma. However, our case has many inadequacies and limitations. First, there was no pathologic biopsy of the cardiac mass to further confirm the diagnosis of lymphoma cardiac infiltrate. In addition, as a case report, its conclusions remain to be confirmed by a large-sample clinical study.

## Data Availability

The original contributions presented in the study are included in the article/Supplementary Material, further inquiries can be directed to the corresponding author.
